# Comprehensive evaluation of AmpliSeq transcriptome, a novel targeted whole transcriptome RNA sequencing methodology for global gene expression analysis

**DOI:** 10.1186/s12864-015-2270-1

**Published:** 2015-12-16

**Authors:** Wenli Li, Amy Turner, Praful Aggarwal, Andrea Matter, Erin Storvick, Donna K. Arnett, Ulrich Broeckel

**Affiliations:** Department of Pediatrics, Section of Genomic Pediatrics, Medical College of Wisconsin, 8701 Watertown Plank Road, Milwaukee, WI 53226 USA; Department of Epidemiology, University of Alabama at Birmingham, 1530 3rd Avenue South, Birmingham, AL 35294 USA

**Keywords:** Targeted gene quantification, Differential gene expression, hiPSC-CMs

## Abstract

**Background:**

Whole transcriptome sequencing (RNA-seq) represents a powerful approach for whole transcriptome gene expression analysis. However, RNA-seq carries a few limitations, e.g., the requirement of a significant amount of input RNA and complications led by non-specific mapping of short reads. The Ion AmpliSeq™ Transcriptome Human Gene Expression Kit (AmpliSeq) was recently introduced by Life Technologies as a whole-transcriptome, targeted gene quantification kit to overcome these limitations of RNA-seq. To assess the performance of this new methodology, we performed a comprehensive comparison of AmpliSeq with RNA-seq using two well-established next-generation sequencing platforms (Illumina HiSeq and Ion Torrent Proton). We analyzed standard reference RNA samples and RNA samples obtained from human induced pluripotent stem cell derived cardiomyocytes (hiPSC-CMs).

**Results:**

Using published data from two standard RNA reference samples, we observed a strong concordance of log2 fold change for all genes when comparing AmpliSeq to Illumina HiSeq (Pearson’s *r* = 0.92) and Ion Torrent Proton (Pearson’s *r* = 0.92). We used ROC, Matthew’s correlation coefficient and RMSD to determine the overall performance characteristics. All three statistical methods demonstrate AmpliSeq as a highly accurate method for differential gene expression analysis. Additionally, for genes with high abundance, AmpliSeq outperforms the two RNA-seq methods. When analyzing four closely related hiPSC-CM lines, we show that both AmpliSeq and RNA-seq capture similar global gene expression patterns consistent with known sources of variations.

**Conclusions:**

Our study indicates that AmpliSeq excels in the limiting areas of RNA-seq for gene expression quantification analysis. Thus, AmpliSeq stands as a very sensitive and cost-effective approach for very large scale gene expression analysis and mRNA marker screening with high accuracy.

**Electronic supplementary material:**

The online version of this article (doi:10.1186/s12864-015-2270-1) contains supplementary material, which is available to authorized users.

## Background

Transcriptome analysis has enhanced our understanding of the molecular constituents of cells and tissues. As one of the most widely used tools for transcriptome profiling, quantification of differential gene expression has played a pivotal role in the identification of pathway and gene-network interactions [1–3]. In the past decade, microarrays and RT-qPCR have been the primary tools for analyzing gene expression changes. Microarray is a hybridization-based approach which typically involves incubation of fluorescence-labeled cDNA with custom-made microarrays or commercially available high-density oligo microarrays [3]. Some specialized microarrays can be used to detect and quantify splice forms [4], and genomic tiling arrays have been constructed to allow the mapping of transcribed regions at the whole-genome level for large genomes with very high resolution [5–7]. Even though these array-based methods are high-throughput, they are physically limited by probe density and suffer from noisy signals resulting from background noise due to cross hybridization and signal saturation [3, 8–10]. RT-qPCR has been considered the gold-standard for gene expression quantification with high accuracy and sensitivity [11]. This method can be used to measure the abundance of a single transcript by measuring fluorescent signal intensity through a real-time PCR system. Though accepted as the gold-standard for measuring gene expression, this method is lower throughput and not amenable to perform global expression analyses. Further, substantial amount of RNA may be required if a large number of genes need to be tested. This imposes some major hurdles for the high-throughput usage of RT-qPCR.

Whole-transcriptome sequencing (RNA-seq) technology has emerged as a revolutionary platform for genome-wide quantification of mRNA transcripts. This technique enables the sequencing of all RNA molecules in a high-throughput manner. Short sequence reads are generated by either single-end or paired-end sequencing. The number of sequencing reads that map to each transcript is used to infer the abundance of mRNA molecules. RNA-seq offers several advantages over microarrays or RT-qPCR for detecting differentially expressed genes (DEGs). These include a wide coverage of transcripts, high sensitivity, the ability to detect allele-specific differential expression and the identification of novel transcripts [12–14]. However, in certain situations RNA-seq may not be a practical choice. For example, since RNA-seq requires a significant amount of starting material, it will not be applicable when only small amount of RNA is available [10]. Further, resolution at a single-base level enabled by RNA-seq may not be necessary if the main goal of a study is to simply assess expression changes on a gene level. For large scale DEG experiments that involve hundreds or even thousands of samples, the bioinformatics analysis as well as data storage needs of RNA-seq become formidable. Further, the complex data sets produced by RNA-seq might limit its use in molecular diagnostic testing, which requires a quick turn-around time for high-quality assessment for DEGs. In these and other situations, a targeted, quantitative RNA-sequencing method with high accuracy and reproducibility can offer a better approach.

Taking advantage of high-throughput sequencing technology in combination with multiplexed enrichment or amplification methods, a few targeted RNA expression kits have recently been developed. For example, Illumina developed TruSeq targeted gene expression kit. This kit enables the testing of 10–100 assays for targeted genes (http://www.illumina.com/products/truseq-targeted-rna-expression-kits.html). Similarly, Thermo Fisher Scientitic developed AmpliSeq-RNA panels for the study of a small number of pre-defined gene sets (between 150 to 900 genes). Based on a number of studies that used a targeted sequencing approach, there is strong evidence that gene expression quantification using targeted sequencing method is reliable and accurate [10, 15]. In light of these findings, a targeted whole transcriptome kit was recently developed (AmpliSeq, Life Technologies AmpliSeq^TM^ technology). This kit is designed for targeted amplification of over 20,000 distinct human RNA targets simultaneously in a single primer pool. A short amplicon (~150 bp) is amplified for each targeted gene [16]. This targeted, high-throughput strategy gives AmpliSeq the advantages of a short turn-around time and a much smaller amount of raw reads required for accurate gene expression quantification than traditional whole transcriptome RNA sequencing.

Here, we performed a comprehensive performance comparison of the AmpliSeq panel using two well-established RNA-seq methods for genome-wide DEG analysis. Our performance assessment for AmpliSeq was conducted on two different sets of RNA samples. First, we tested two commonly used, commercially available reference RNA samples. This allowed us to take advantage of established expression data from various analysis platforms. Our analysis consistently suggests that AmpliSeq performs as well as RNA-seq for gene expression quantification. Second, we provide further assessment of AmpliSeq using RNA samples from human induced pluripotent stem cell derived cardiomyocytes (hiPSC-CMs). In comparison to the reference samples, these lines exhibit a spectrum of expression patterns, which can be expected in typical expression studies. Notably, hiPSC-based cell lines are gaining popularity as significant tools for disease modeling and comprehensive functional analysis, e.g., the analysis of gene expression influenced by human genome variation and validation of functional impact of a causal variant. In addition to correlating the fold-change in expression, we also used clustering and principal component analysis to compare the overall performance of AmpliSeq to RNA-seq in identifying distinct global expression patterns according to known source of variations. AmpliSeq offers comparable resolution to RNA-seq by clustering cell lines based on their origin, phenotypic and disease status. Our study strongly suggests that AmpliSeq is a highly reliable tool for gene expression quantification as demonstrated by the analysis of both reference libraries and real life samples such as iPSC disease models.

## Methods

### Library preparation methods

For the three gene expression quantification methods we compared in this study, three library preparation methods are used: poly-A enrichment for Illumina RNA-seq, ribosomal RNA depletion for Proton RNA-seq, and genome-wide, targeted amplicon amplification for AmpliSeq. For the library preparation, both Illumina and Proton RNA-seq are similar: RNA is subjected to fragmentation prior to random amplification and down-stream high-throughput next-generation sequencing. Both methods have a certain percentage of reads mapped to intronic regions [17].

The major difference between AmpliSeq and the two whole transcriptome RNA sequencing methods is that AmpliSeq is designed to profile over 20,000 distinct human RNA targets using a highly multiplexed amplification method. Each amplicon represents a unique targeted gene. The average size of each amplicon is ~150 bp. Because of the targeted nature and small amplicon size, the total number of raw reads needed for DEG analysis for each library prepared with AmpliSeq is much smaller than typical whole-transcriptome RNA sequencing. For library preparation, a barcoded cDNA library is first generated with SuperScript® VILO™ cDNA Synthesis kit from 10 ng of total RNA. Then cDNA is amplified using Ion AmpliSeq™ technology to accurately maintain expression levels of all targeted genes. Amplified cDNA Libraries were evaluated for quality and quantified using Agilent Bioanalyzer High sensitivity chip. Libraries were then diluted to 100pM and pooled equally, with eight individual samples per pool. Pooled libraries were amplified using emulsion PCR on Ion Torrent OneTouch2 instruments (OT2) and enriched following manufactures instructions. Templated libraries were then sequenced on Ion Torrent Proton™ sequencing system, using Ion PI kit and chip V2.

### Sequencing methods

Two established next-generation sequencing platforms were involved in this study: Illumina RNA-seq and Proton RNA-seq. Both the Proton RNA-seq and AmpliSeq rely on Ion Proton for the sequencing step, though each uses different RNA library preparation protocols (as mentioned above). Illumina RNA-seq and Proton RNA-seq use two next-generation sequencing technologies. Illumina RNA-seq uses the technology of sequencing by synthesis, which requires fluorescence and signal scanning. For this method, prepared libraries are denatured to single strands by linearization. The four nucleotides (GCAT) are coupled to a cleavable fluorescent dye and a removable blocking group, which complements the template one base at a time, yielding a signal to be captured by a charge-coupled device [18]. Proton RNA-seq is based on semiconductor sequencing technology with emulsion PCR amplified libraries bound to Ion spheres. The template spheres are loaded on a sequencing chip and run on the Proton instrument. The sequencing is then performed by flushing nucleotides individually over the surface of the library-loaded chip. Reads are produced by detecting the change in pH as each nucleotide is incorporated [18, 19]. In this study, raw reads from Illumina RNA-seq are pair-ended and those from Proton RNA-seq are single-ended.

### Reference RNA samples

For initial performance analysis, we used two commercially available reference RNA samples. The Agilent Universal Human Reference RNA (UHRR) (reference rna cat. #: #740000) was a pooled sample from 10 cancer cell lines*;* and the Ambion FirstChoice® Human Brain Reference Total RNA (HBRR) was generated from multiple brain regions from 23 donors [20] (reference rna cat. #: #6050). UHRR and HBRR have been previously used for accuracy assessment of various platforms, including microarray, targeted RNA-sequencing using multiplex-PCR amplicons [15] and RNA-seq [17, 20, 21]. In addition, these samples have also been used for evaluating various analytical methods for DEG analysis [22]. In this study we performed AmpliSeq on both UHRR and HBRR samples. Illumina RNA-seq data for UHRR and HBRR was obtained through published data [22] (GEO accession number: GSE49712). Proton RNA-seq data for these two reference RNA samples were obtained from the Association of Biomolecular Resource Facilities next-generation sequencing (ABRF-NGS) study on RNA-seq [17] (GEO accession number: GSE46876). Further, we utilized these RNA-seq datasets to measure the specificity and sensitivity of each gene quantification method examined in this study. All analyses were conducted on three replicates of each sample.

We performed AmpliSeq on both UHRR and HBRR samples. For quality control, ten External RNA Controls Consortium (ERCC) [23] reference materials (Life Technologies) were added to each RNA sample in both the UHRR and HBRR RNA samples prior to library construction. The ERCC spike-in controls used in this study are comprised of 10 synthetic polyadenylated oligonucleotides, with lengths varying from 250 to 2000 bp and input concentrations ranging from 0.45 to 1875 attomoles/*μ*l (which translates to a log2 concentration of −1.12 to 11.87).

### RNA samples obtained from hiPSC-CMs

We assessed the performance of AmpliSeq in comparison to Proton RNA-Seq using four biologically related RNA samples. We selected RNA from two patient-specific hiPSC-CM lines. These two lines are selected from participants in the NHLBI HyperGen cohort [24, 25]. This study has been approved by the review boards of all participating institutions (University of Alabama at Birmingham’s Institutional Review Board for Human Use, University of Utah’s Institutional Review Board, and Medical College of Wisconsin’s Institutional Review Board). All human subjects in this study gave informed consent.

These two cell lines differ genetically as well as phenotypically in various echocardiographic measurements such as left ventricular mass and ejection fraction. The hiPSC lines were generated from white blood cells using episomal reprogramming [26]. hiPSC-CMs were obtained from a single batch of cells. These hiPSC-CMs are highly pure (>95 % purity) cardiomyocytes with functional properties resembling adult human CMs [27]. The hiPSC-CMs were maintained in iCell Maintenance Medium (iCMM) (Cellular Dynamics International, Madison, WI, US). The hiPSC-CMs were stimulated with endothelin-1 (ET-1) to induce a hypertrophic response following our established CM hypertrophy protocol [28]. Cardiomyocytes were harvested at both18 h post stimulation and before stimulation with Total RNA Purification 96-Well Kit (Norgen Biotek Corp.). Total RNA was extracted per manufacturer’s recommendations, and re-suspended in nuclease-free water. The RNA used for library preparation was then further concentrated using Qiagen RNeasy MinElute Cleanup Kit (Qiagen) and quantified by UV spectrophotometry (NanoDrop™ 2000, Thermo Scientific). Hypertrophy was confirmed by analysis of several cardiac hypertrophy markers, including *NPPB*. For this study, we performed both AmpliSeq and RNA-seq on the Ion Proton platform for both stimulated and unstimulated hiPSC-CMs.

### Read alignment and differential gene expression analysis

Raw reads from all whole transcriptome RNA-seq libraries were aligned using a two-step alignment approach. First, TopHat (v.2.0.3) [29] was used with the following settings: ‘-r 70 --mate-std-dec 90′ for paired-end reads from Illumina RNA-seq; and ‘-r 200’ for single-end reads from Proton RNA-seq. Second, unmapped reads from step one were realigned with Bowtie2 [30] using the “–very-sensitive-local” method. The genome annotation (GTF) file generated by the UCSC genome browser was used as reference. Genes shorter than 150 bp were excluded from the GTF file. Raw reads shorter than 50 bps were excluded from the alignment process. RNA-seq reads aligned using the two-step approach include published Illumina and Proton RNA-seq data for UHRR and HBRR samples, and Proton RNA-seq data for in-house hiPSC-CM samples. Raw read counts for each gene were obtained using HTSeq (v0.6) [31]. Combined (Tophat + bowtie2) sequence alignment generated by the two-step alignment approach served as input file for HTSeq.

Primary analysis for AmpliSeq sequencing data of all samples was performed using the ampliSeqRNA plugin available for Ion Torrent™ sequencing platforms. This plugin uses the Torrent Mapping Alignment Program (TMAP). TMAP is optimized for Ion Torrent™ sequencing data for aligning the raw sequencing reads against a custom reference sequence set containing all transcripts targeted by the AmpliSeq kit. To maintain specificity and sensitivity, TMAP implements a two-stage mapping approach. First, four alignment algorithms, BWA-short [32], BWA-long [33], SSAHA [34], and Super-maximal Exact Matching [35] we employed to identify a list of Candidate Mapping Locations (CMLs). A further aligning process is performed using the Smith Waterman algorithm [36] to find the final best mapping [37]. As part of the ampliSeqRNA plugin, raw read counts of the targeted genes is performed using samtools (samtools view –c –F 4 –L bed_file bam_file).

DEG analysis was performed using R/Bioconductor package DESeq2 [1] with raw read counts from RNA-Seq and AmpliSeq. Read count normalization was performed using the regularized logarithm (rlog) method provided in DESeq2. Genes with less than ten normalized read counts were excluded from further analysis. DEGs were determined by *p-*value and the log2 fold change (log2FC) by DESeq2.

### Performance assessment using RT-qPCR validated gene sets as gold-standard

Two published RT-qPCR datasets for DEGs between the UHRR and HBRR library were used as gold standard to assess the overall performance of each method. One dataset is comprised of TaqMan assays for 843 genes from the MAQC-I study (MAQC) (GSE5350) [20]. The other dataset uses PrimePCR to quantify 20,801 genes as part of the ABRF-NGS consortium [21]. All PrimePCR assays have been validated extensively [17]. The C_T_ value of each target was normalized by subtracting the average C_T_ of endogenous control from the C_T_ of each RT-qPCR target.

The overall concordance between each of the interested method and RT-qPCR was measured by calculating the Pearson’s r on individual gene’s log2FC values. To measure the similarity of each method in ordering the expression of genes, we calculated Spearman’s ranked r between each method using log2 transformed read counts. Three statistical analyses were used to compare transcript FC detected by each of the three methods to the gold-standard qPCR data: root-mean-square deviation (RMSD) for genes with at least two FC in expression, Receiver Operator Characteristic curves (ROC curve) with their associated Area Under the Curve (AUC) value, and Matthew’s correlation coefficient (MCC).

Using the ABRF gene set as the gold-standard, we calculated the RMSD for genes with at least two FC in expression. This statistical analysis provided a quantitative measure of the difference in the identified FC of each method to RT-qPCR standard [22]. We set a FC cutoff of two for this analysis. To further assess the potential influence of transcript abundance, we calculated the RMSD values for gene sets grouped by normalized read counts. Transcripts were grouped starting at 15 normalized read counts as determined by DESeq2 with increments of 20. This analysis is based on the read counts measured by the Illumina RNA-seq data.

We used AUC and MCC to measure the overall specificity and sensitivity of each method. Higher AUC values indicate better overall accuracy. It is perceivable that transcript abundance influences the ROC characteristics. Therefore, we assessed the impact of transcript abundance by categorizing transcript levels into quartiles based on Illumina RNA-seq data. ROC analysis was performed in comparison to the ABRF PrimePCR dataset. Similar to the study of Li et al. [17], we calculate the AUC of the ROC curve for each quartile. The ROC metric from Python scikit-learn [38] was used to construct ROC curves and calculate associated AUC values. Additionally, we used MCC as another method for measuring overall accuracy. Based on Illumina RNA-seq data, we determined the significance level of differential gene expression by DESeq2. Using the same *p*-value cutoffs as described by Li et al. (2014), transcripts were then grouped by *p*-values at 0.05, 0.01 and 0.001. Only transcripts with at least two-fold change were included in the analysis.

### Comparison between AmpliSeq and Proton RNA-Seq using hiPSC-CM lines

In addition to the standard reference libraries, we also analyzed four RNA samples taken from two patient-specific, hiPSC-derived CM lines. Both cell lines were stimulated with ET-1 to induce a hypertrophic phenotype [28]. The stimulated as well as unstimulated RNA samples from each cell line were prepared and sequenced using both the AmpliSeq and Proton RNA-seq on the Ion Torrent Proton. DEGs after stimulation were filtered by at least 10 normalized read counts and an FC cutoff of two. For these genes, Pearson’s r was calculated between log2FC measured by Proton RNA-seq and AmpliSeq. Only annotated features common in both platforms were included for further analysis. Hierarchical clustering and principal component (PCA) analyses were used to study the performance of AmpliSeq and Proton RNA-seq in identifying distinct global expression patterns between the cell lines and the impact of the experimental conditions. To remove any batch effects due to the substantial sequencing depth differences between the RNA-Seq and AmpliSeq libraries, we used the removeBatchEffect function from the R/Bioconductor package limma [39]. The batch effect removal was performed on the regularized logarithm transformed read counts for all the hiPSC libraries. Hierarchical clustering was performed using the hclust functions in R. Spearman correlation (cor function in R) matrix based dissimilarity (1 – correlation) was provided as the distance metric. PCA was performed using all genes common to both Proton RNA-seq and AmpliSeq.

## Results

### Technical reproducibility and consistency of AmpliSeq runs

When preparing the libraries for AmpliSeq, ten ERCC transcripts with known concentrations were spiked in for each technical run before library preparation of UHRR and HBRR samples. These transcripts have a nearly 2000 fold difference regarding transcript abundance. The detected abundance of ERCC transcripts helped us evaluate the quality of the sequenced UHRR and HBRR libraries. Values of Pearson’s r were calculated for the following: (1) The overall concordance between detected read-counts and known concentrations of each ERCC transcript using log2 transfromed data; and (2) the overall concordance of read-counts of all genes between replicates using log10 transformed raw read-counts.

**Fig. 1:**
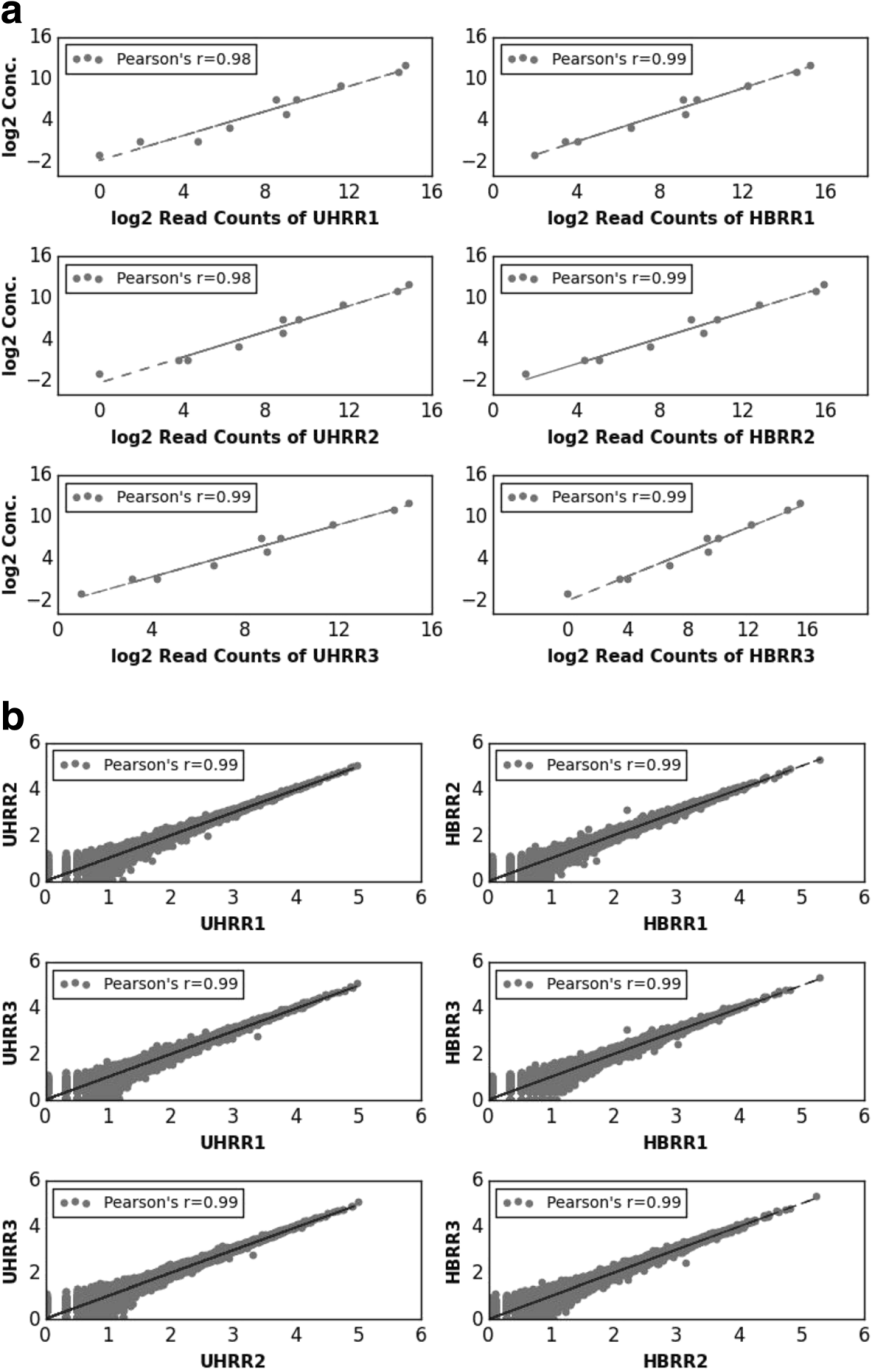
Technical reproducibility and consistency of AmpliSeq runs based on ERCC spike-in control analysis. **a** Correlations between known concentration of ERCC spike-in controls and detected read-counts of AmpliSeq. Pearson’s *r* values are 0.98 for each comparison. **b** When comparing each of the technical replicates against each other, Pearson’s *r* values are 0.99 for each comparison

The values of Pearson’ r between the log2 transformed known concentration and detected read-counts are > = 0.98 (Fig. 1a), indicating high-quality sequencing. We observed minimal technical variations among the technical replicates of each sample. Pearson’ r of log10 transformed raw read-counts of all genes between the three technical replicates of UHRR and HBRR are =0.99, indicating very consistent performance between technical replicates (Fig. 1b).

### General performance summary for AmpliSeq, Illumina RNA-seq and Proton RNA-seq

For the published RNA-seq data, 40 million reads per sample were obtained for both Illumina RNA-seq and Proton RNA-seq. Due to its targeted nature and short length of each targeted amplicon, an average of nine million reads was obtained for each sample with each AmpliSeq run (GEO accession #: GSE74760). Vast majority of the reads (> = 93.2 %) were on target for each run (Additional file 1: Table S1).

Using DESeq2 normalized transcript read counts (normalization procedure described in methods) from Illumina RNA-Seq, we observe a nearly equal distribution in each quartile of transcript abundance for genes with at least two FC in expression (Additional file 1: Figure S1). AmpliSeq, Illumina RNA-seq and Proton RNA-seq respectively identified 12081, 14,222, and 12,205 genes with FC > =1.5; and 9287, 11,954 and 9220 genes with FC > =2. With FC = 1.5 as the cutoff, AmpliSeq identified 68 % and 72 % of the genes identified by Illumina RNA-seq and Proton RNA-seq respectively. With FC = 2 as the cutoff, AmpliSeq identified 65 % of the genes identified by Illumina RNA-seq and 69 % by Proton RNA-seq.

For our hiPSC-CM samples, Proton RNA-seq generated an average of 44 million reads for each sample. Out of all the reads generated, 14.9 % to 17.5 % of the reads are due to duplicated reads (GEO accession #: GSE74760). Using Gencode gtf as the reference, the mapping rate for each sample is between 93.2 % and 94.6 % (Additional file 1: Table S2). For each AmpliSeq run, an average of 10 million reads was generated for each sample. For each sample, > = 94 % of the reads are on target (Additional file 1: Table S3).

**Fig. 2:**
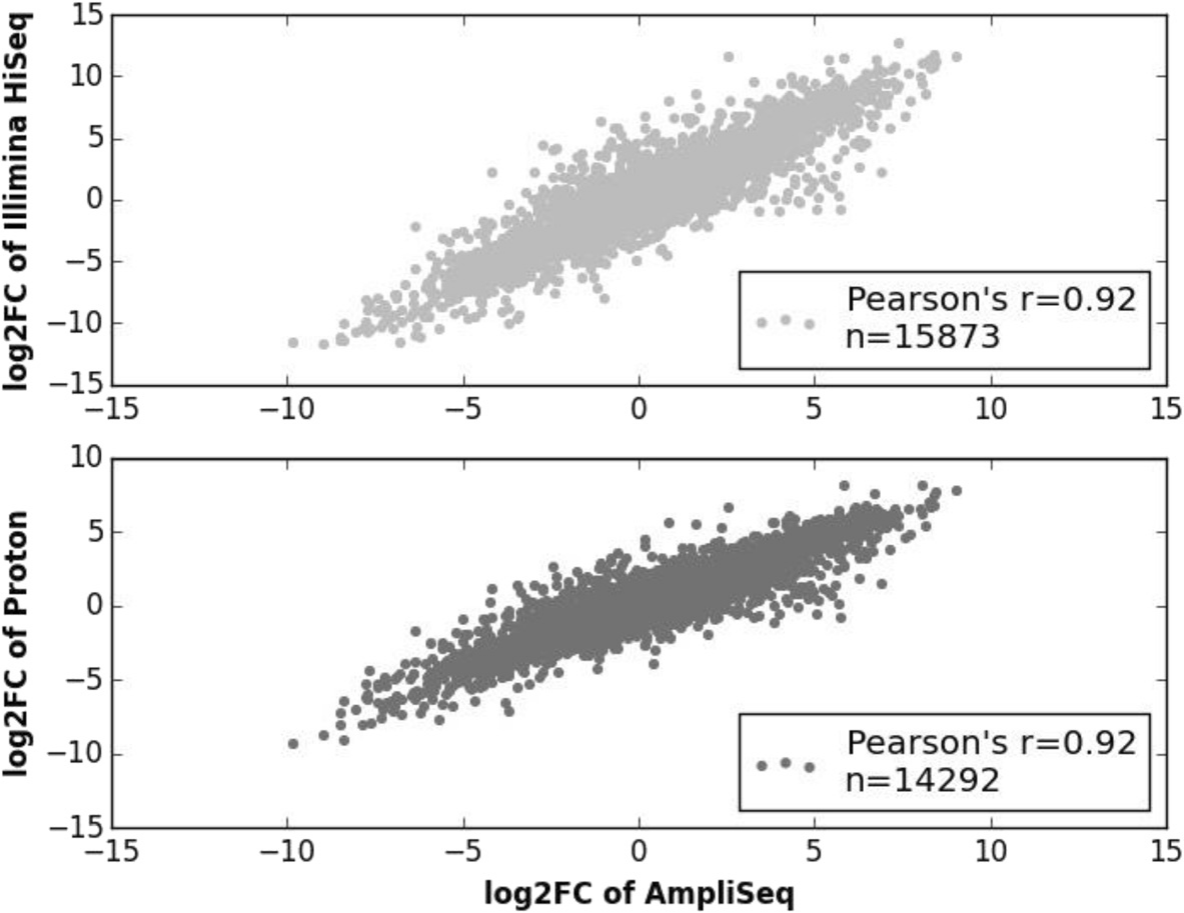
High correlations of log2FC were observed between both RNA-seq methods and AmpliSeq for all genes*.* The Pearson’s *r* values are 0.92 for Illumina RNA-seq vs*. AmpliSeq* (*top panel*, *n* = 15,873) and Proton RNA-seq vs. AmpliSeq (*bottom* *panel*, *n* = 14,292)

Using log10 transformed normalized read counts for RNA reference samples, we calculated Spearman ranked correlation in the pairwise comparison of the three methods. The strong Spearman’s r values indicate similar ability of each method in ordering the expression genes (values of Spearman ranked *r* > =0.80) (Additional file 1: Figure S2A and B). We further calculated the correlation of log2FC between both RNA-seq methods and AmpliSeq of all genes for RNA reference samples. The Pearson’s *r* values are 0.92 for both Illumina RNA-seq vs. AmpliSeq (Fig. 2, top panel) and Proton RNA-seq vs. AmpliSeq (Fig. 2, bottom panel). For our hiPSC-CMs samples, we observed significant correlation between Proton RNA-seq and Ampliseq using log10 transformed read-counts and (Additional file 1: Figure S3) log2FC for all genes (Additional file 1: Figure S4). These correlation analyses indicate highly significant correlation between AmpliSeq and the two RNA-seq methods. However, correlation values do not indicate overall accuracy of the methods compared in this study.

### AmpliSeq has comparable accuracy to Illumina RNA-seq and Proton RNA-seq for DEGs between UHRR and HBRR

Overall, all three methods show excellent correlation against the RT-qPCR results for all genes in terms of log2FC. Using the MAQC dataset as the standard, we observe Pearson’s *r* values of 0.95 between the log2FC determined by AmpliSeq and the two RNA-seq methods. For the ABRF PrimePCR dataset, the Pearson’s values were > =0.89 (Additional file 1: Figure S5).

**Fig. 3:**
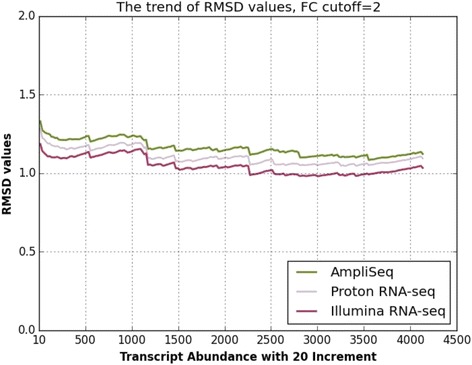
RMSD values of the three methods are in close range with each other for genes with at least two FC in expression

RMSD values of the three methods are highly comparable for genes with at least two FC (Fig. 3). For all the analyzed transcripts, the average RMSD values are highly similar (1.16 ± 0.05 (AmpliSeq, mean ± s.e.), 1.1 ± 0.05 (Proton RNA-seq, mean ± s.e.) and 1.05 ± 0.05 (Illumina RNA-seq, mean ± s.e.)). This suggests that AmpliSeq exhibits an equal ability to detect and measure expression levels of DEGs in comparison to the two RNA-seq methods.

**Fig. 4:**
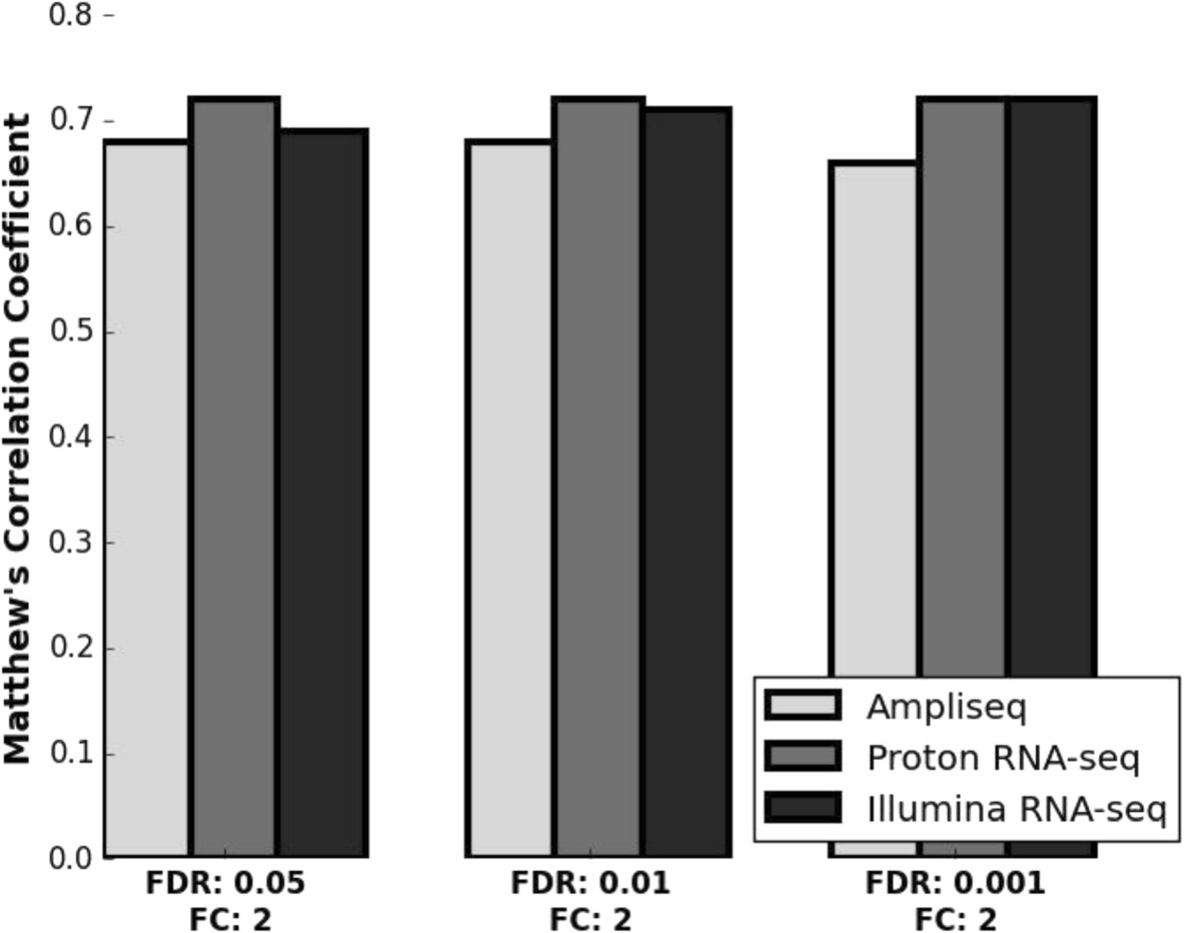
Using the MAQC dataset as the gold-standard, MCC values from Illumina RNA-seq, Proton RNA-seq and Ampliseq are in close range with each other for the three *p*-value cutoffs (0.05, 0.01 and 0.001) used

**Fig. 5:**
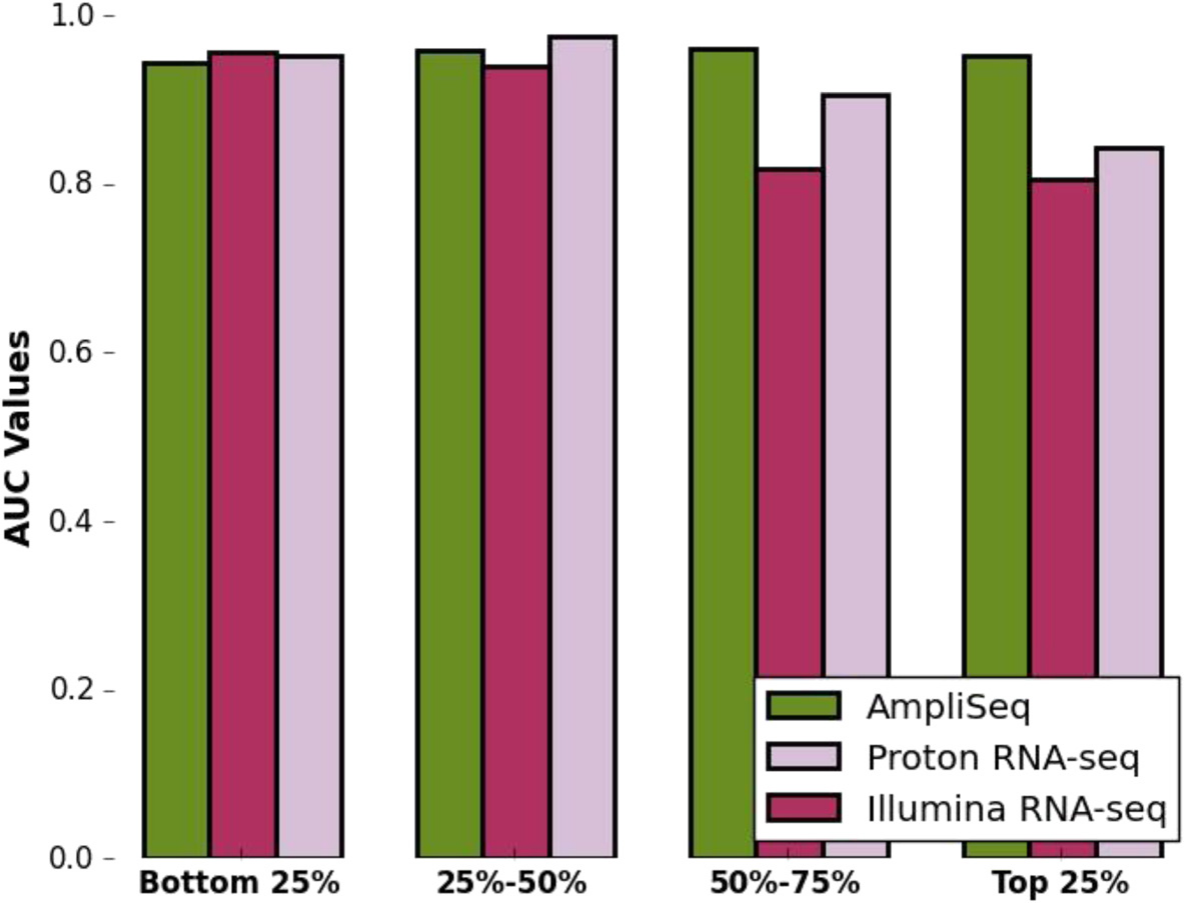
AmpliSeq is robust in identifying significantly differentially expressed genes between UHRR and HBRR using the ARBF PrimePCR as the gold-standard. For genes in the two bottom quartiles, AmpliSeq performs comparably to the two RNA-seq methods. The average AUC values for AmpliSeq, Illumina RNA-seq and Proton RNA-seq are 0.95, 0.96 and 0.95 respectively. For genes in the top two quartiles, AmpliSeq performed better than the two RNA-seq methods with an average AUC value of 0.95 while Illumina and Proton RNA-seq have an average of 0.81 and 0.84 respectively

Using the MAQC dataset as the gold-standard, MCC values are in close range with each other indicating that all three methods are comparable in terms of accuracy for each of the three *p*-value cutoffs (0.05, 0.01 and 0.001) (Fig. 4). To further assess how transcript abundance affects the ability of each method to identify truly differentially expressed genes, we used the ABRF PrimePCR as the standard. We divided the genes into four subgroups based on three quartile points of normalized read-counts as determined by the Illumina RNA-seq data. Subsequently, we calculated the AUC values for each quartile. For genes in the two bottom quartiles, AmpliSeq performs comparably to the two RNA-seq methods. The average AUC values for AmpliSeq, Illumina RNA-seq and Proton RNA-seq are 0.95, 0.96 and 0.95 respectively. For genes in the top two quartiles, AmpliSeq performed better than the two RNA-seq methods (Fig. 5) with an average AUC value of 0.95; Illumina and Proton RNA-seq have a average AUC of 0.81 and 0.84 respectively. In summary, all the analysis performed in this study indicated that that AmpliSeq is very robust in identifying significantly differentially expressed genes between UHRR and HBRR, and it has a better performance for transcripts that fall into the upper-half abundance spectrum.

### AmpliSeq yields similar resolution as Proton RNA-seq for RNA samples obtained from hiPSC-CMs

Analysis using UHRR and HBRR provided valuable insights about the performance of AmpliSeq. However, UHRR and HBRR are pooled samples from multiple sources. Therefore, the differential gene expression profile may not represent the typical RNA samples in a research or clinical setting. Consequently, we extended our analysis and analyzed four biologically relevant samples. These samples were obtained from two different hiPSC-CM lines allowing us to further assess the performance of AmpliSeq in comparison to the Proton RNA-Seq. Since the two individuals, from which the hiPSC-CMs were derived, differ in genetic as well as phenotypic features, we expect to detect different, cell-line specific gene expression profiles.

As expected, different set of DEGs were identified for samples 1156 and 1104. 67 % of all DEGs identified in sample 1156 were shared by sample 1104, and 24 % of DEGs identified in sample 1104 were shared by sample 1156. Though a largely different set of genes showed significant differential expression for each sample, we observed strong correlation of the log2FC between AmpliSeq and Proton RNA-seq for both CM lines (Pearson’s *r* = 0.96 for genes with FC > =2; and Pearson’*r* > =0.90 for genes with FC > =1.5). Thus, AmpliSeq has very consistent performance compared to Proton RNA-seq in identifying differentially expressed genes when closely related RNA samples were used.

**Fig. 6:**
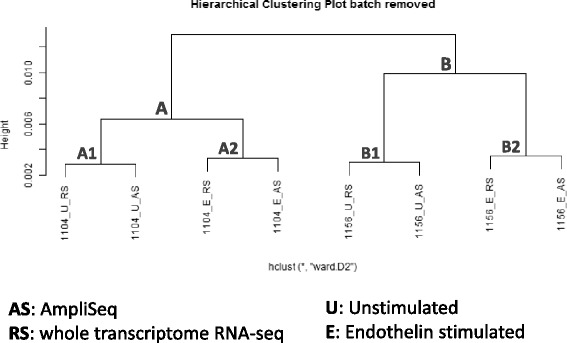
AmpliSeq offers the same ability as Proton RNA-seq in capturing global gene expression patterns that is consistent with known source of variations. All samples clustered into two groups based on the origin of hiPSCs (Groups *a* and *b*), reflecting the impact of genetic variation. Within each group, samples separate into the stimulated and unstimulated subgroups (*A1* and *A2*; *B1* and *B2*). Further, the samples separate based on the respective sequencing methods used. AS for AmpliSeq, RS for RNA-seq, unstim for unstimulated, ET-1 for Endothelin 1 stimulated

**Fig. 7:**
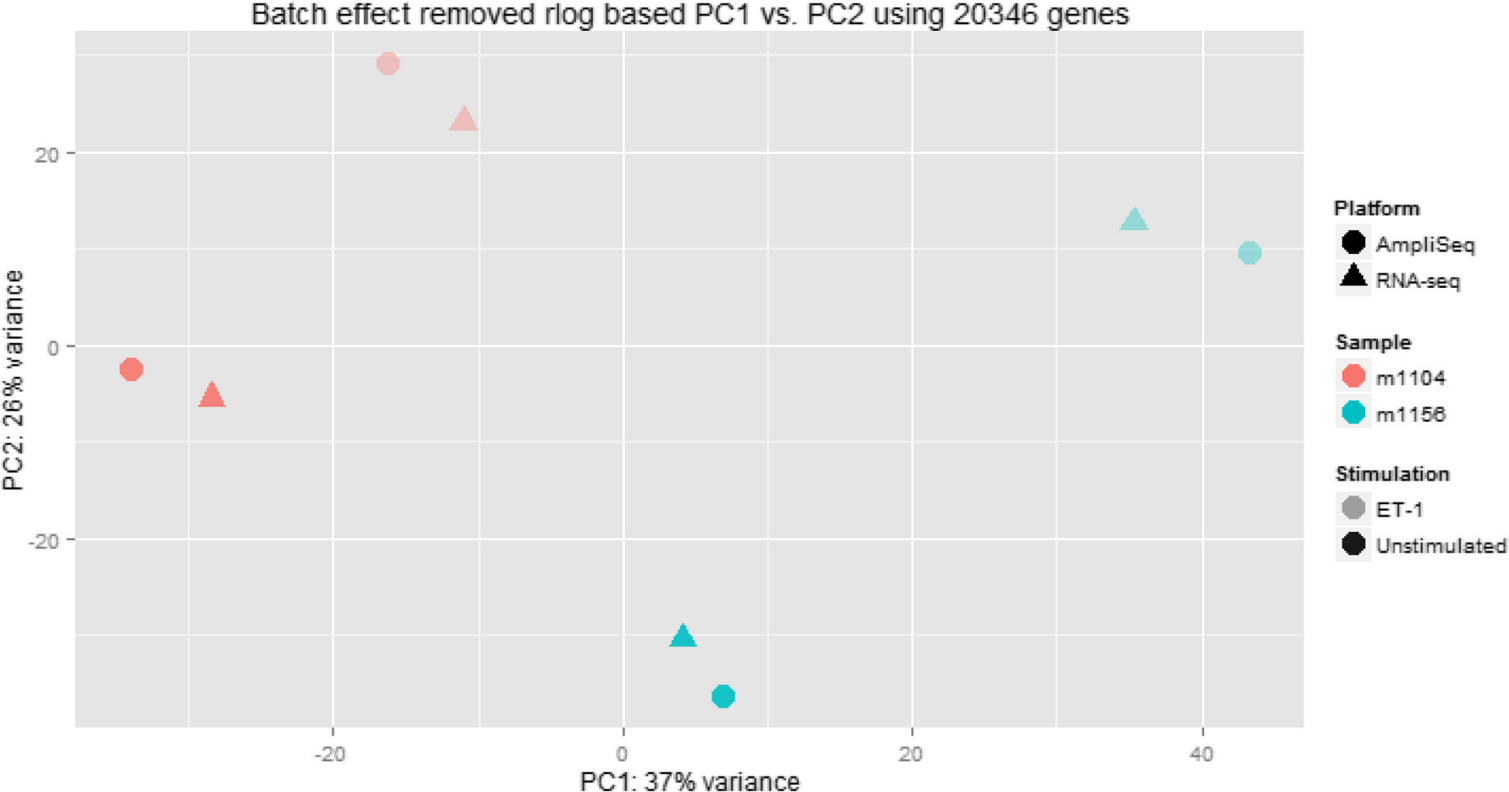
Principal component analysis (PCA) analysis using all genes common to both AmpliSeq and Proton RNA-seq. PCA identifies the differences between the donors of hiPSC lines as principal component 1 (PC1), and the variation due to stimulation as PC2. For platform labeling, solid circle indicates AmpliSeq and solid triangle indicates RNA-seq. For hiPSC-CM samples, hiPSC donor 1104 is in orange and hiPSC donor 1156 is in blue with light shade indicating ET1-stimulated sample and dark shade indicating unstimulated sample

Furthermore, we used both clustering and PCA analysis to assess the performance of AmpliSeq and Proton RNA-seq in identifying distinct global expression patterns based on known source of variations between the cell lines. We expect to observe three distinct sources of variations in our experimental design: (1) differences due to the genetic makeup of hiPSC donors; (2) differences based on hypertrophy phenotypes, non-hypertrophic (unstimulated) vs. hypertrophic (stimulated) cells, and (3) differences associated with the library preparation methods: targeted amplification for AmpliSeq and random amplification of ribo-depleted RNA library for Proton RNA-seq. As can be seen in the dendrogram in Fig. 6, AmpliSeq offers the same ability as Proton RNA-seq in capturing these variations at global gene expression level. As expected, all samples clustered into two groups based on the origin of hiPSCs (Groups A and B), indicating the biggest source of variation in expression difference is introduced by the cell line itself, reflecting the impact of genetic variation. Within each group, samples separate into the stimulated and unstimulated subgroups (A1 and A2; B1 and B2). Further, the samples also separate based on the respective sequencing methods used (AS and RS). As an independent method, PCA analysis shows the same pattern as clustering analysis. Principal component 1 (PC1) represents the differences between the donors of hiPSC lines as the largest source of variation. Principal component 2 (PC2) represents the variation due to stimulation (Fig. 7). Importantly, these results are consistent and can be observed in both clustering and PCA analyses.

## Discussion

### AmpliSeq transcriptome offers a robust method for large-scale genome-wide differential gene expression analysis

Whole transcriptome sequencing has emerged as a new standard for high-throughput transcriptomic studies [17, 21, 40]. This powerful method is robust and versatile for quantitative measurements of gene expression, identification of splice variants, single nucleotide polymorphisms (SNPs), and discovery of novel protein-coding genes by *de novo* assembly. However, for research focused on studying differential expression of known genes, sequence information at single-base level may not be required, thus motivating the development of alternative methods for high-throughput, yet highly accurate quantification of gene expression. Further, there are few well-recognized limitations for the application of whole-transcriptome RNA-seq. At the time of our analysis, whole-transcriptome sequencing methods require a significant amount of input material. To ensure sufficient statistical power, studies of functional consequences of differential gene expression generally entail large scale of cohorts and multiple replicates of each sample. Such requirement can easily lead to formidable sequencing cost and significant efforts for downstream analysis. After sequencing, conventional work-flow of RNA-seq involves mapping short reads to genomic reference, which unavoidably involves the mapping of non-specific in highly repetitive regions [41]. Such non-specific read-mapping can cause complications in transcript quantification.

AmpliSeq, as a whole-transcriptome, targeted gene quantification kit, seems to excel in all these limiting areas of RNA-seq. With 10 ng of total RNA, minimal prep time and the requirement of fewer sequencing reads compared to RNA-seq, AmpliSeq displayed very robust performance without losing specificity and sensitivity. First, results from ERCC spike-in controls in our AmpliSeq run of UHRR and HBRR indicated that AmpliSeq is highly consistent and yields high-quality transcript quantification. Despite the wide range in defined transcript abundance (in a range of 2000 fold) for the ERCC spike-in controls, detected read-counts and known concentrations of each ERCC transcript are highly correlated, reflecting great sequencing quality of AmpliSeq. By comparing the raw read-counts of all genes between replicates, we observed a very consistent performance between technical replicates. Second, our analysis using the DEGs between two reference libraries indicated that the performance of AmpliSeq closely resembles established RNA-seq methods in terms of overall specificity and sensitivity as indicated by the values of RMSD, AUC and MCC. Notably, AmpliSeq displayed excellent accuracy for genes in bottom two quartiles and outperforms the two RNA-seq methods for genes with high abundance. Third, even though AmpliSeq does not have capacity in isoform-level quantification or resolution at single-base level, sequencing-based, targeted quantification gives AmpliSeq methodology an advantage in handling off-target amplification. By only considering reads matching to defined target regions, AmpliSeq reduces the complexity and could avoid issues related to non-specific mapping [10].

As a new method for sequence-based, genome-scale gene expression quantification, AmpliSeq stands as a very versatile and cost-effective approach for large-scale gene expression analysis with high accuracy. These merits enable AmpliSeq as a highly attractive method for very large-scale studies including replication analysis or other studies that require a very large number of samples to increase statistical power.

### AmpliSeq is competent at capturing global gene expression patterns in hiPSC-CM RNA samples based on defined source of variation

One concern of using the standard RNA samples for quality assessment from various platforms for DEG analysis has been the distinct gene expression profile between the two reference RNA samples. Both UHRR and HBRR samples are pooled samples either from different cancer cell lines or from several regions of the brain from multiple donors. Additionally, more than 50 % the genes in human brain have brain-specific expression, which makes HBRR drastically different from UHRR [20, 42, 43]. Thus, the uniquely expressed genes in each sample did not represent the expression profiles in a typical biological experiment involving physiologically relevant RNA samples [42].

In order to gain insights into the performance of AmpliSeq to an established RNA-seq method, we used samples which represent expression patterns more commonly observed in a typical experiment. We performed AmpliSeq on four RNA samples obtained from an in vitro model of cardiac hypertrophy [28]. The hiPSC-based in vitro cellular model is gaining prominence in the fields of drug screening and disease modeling. Such a model enables in vitro generation of human tissue types, which allows patient-specific assays for functional interrogation of genetic variants or expression profiling of disease relevant genes. We have previously shown that the expression pattern of hiPSC-CMs resembles the expression patterns observed in human myocardial biopsies [28].

Comparing Proton RNA-seq and AmpliSeq results for the four RNA samples, we observe nearly the same clustering pattern between the two platforms. Out of the three levels of variations predefined by our experiments, we expect hiPSC-donors to be the largest variation, followed by hypertrophy phenotype and with library preparation methods being the least variation that contribute to global gene expression pattern. As expected, AmpliSeq, just like RNA-seq, is able to capture these variations shown in the clustering analysis. Both methods first differentiate the patient-specific cell lines, and then subsequently separate them based on their hypertrophy phenotype. Most importantly, AmpliSeq achieves the same resolution as RNA-seq with much less number of reads. An independent method, PCA, also revealed the same conclusion. Combined with our findings from UHRR and HBRR samples, our results strongly support that AmpliSeq is a very sensitive and competent approach for very large-scale mRNA-marker screening in hiPSC-based cellular models.

### The need for the establishment of gold-standard RNA dataset from more diversified conditions

Our understanding of the complexity and diversity of the human transcriptome is far from being comprehensive. Hence, there is a clear need to develop more complex RNA samples with well-validated RNA content that are from a wide range of physiological conditions. Create and analyze a very comprehensive RNA dataset requires major effort like MAQC III [44, 45] and GEUVADIS [46]. The establishment of complex, standard RNA samples/datasets and their associated RNA content verification will undoubtedly help the maturity of new technologies and statistical metric for transcript quantification. Amplification based methods such as the AmpliSeq method clearly can have a significant contribution in this overarching goal based on the performance we have observed in this study.

## Conclusions

AmpliSeq, as a whole-transcriptome, targeted gene quantification method, clearly stands as a highly robust approach for large-scale, genome-wide differential gene expression analysis. Further, AmpliSeq is a very sensitive and competent approach for very large-scale mRNA-marker screening in cellular models.

### Availability of supporting data

The data sets supporting the results of this article are available in the NCBI Gene Expression Omnibus (GEO) repository, GSE74760. This includes AmpliSeq raw read counts on both RNA reference samples and hiPSC-CM samples, and Proton RNA-seq raw read counts on hiPSC-CM samples.

## Additional file

Additional file 1: Figure S1. Genes with at least two-fold change in expression between UHRR and HBRR have a nearly even distribution in four quartiles in terms of transcript abundance based on normalized transcript read-counts from Illumina RNA-seq. **Figure S2.** a.) Spearman’s ranked r for all genes using log10 transformed read-counts. AmpliSeq showed a strong correlation to the two whole transcriptome RNA-seq methods as determined by Spearman’s ranked r. b.) Dotplots of gene expression between different sequencing platforms. **Figure S3.** Significant correlation (*p* < 1e-6) of gene expression (using log10 transformed read-counts) between AmpliSeq and Proton RNA-seq for the following samples: hiPSC-CM 1104 at stimulated condition, hiPSC-CM 1104 at unstimulated condition, hiPSC-CM 1156 at stimulated condition and hiPSC-CM 1156 at unstimulated condition (E: Endothelin 1 stimulated, U: unstimulated). **Figure S4.** Significant correlation (*p* < 1e-6) of log2FC between AmpliSeq and Proton RNA-seq for samples hiPSC-CM 1156 (ET vs. unstim, *n* = 10,183) and hiPSC-CM 1104 (ET. vs. unstim, *n* = 10,226). **Figure S5.** All three methods show strong correlation against the RT-qPCR results in terms of log2FC. Using the MAQC dataset as the standard, we observe Pearson’s *r* values of 0.95 between the log2FC determined by AmpliSeq and the two RNA-seq methods (*n* = 674). For the ABRF PrimePCR dataset, the Pearson’s values were > =0.89 (*n* = 13,747). (ZIP 295 kb)

## Abbreviations

AmpliSeq: Ion AmpliSeqTM Transcriptome Human Gene Expression Kit
AUC: area under the curve
DEGs: differentially expressed genes
ERCC: External RNA Controls Consortium
GTF: genome annotation file
HBRR: Ambion FirstChoice® Human Brain Reference Total RNA
hiPSC-CMs: human induced pluripotent stem cell derived cardiomyocytes
Log2FC: log2 fold change
MCC: Matthew’s correlation coefficientTargeted gene quantification, Differential gene expression, hiPSC-CMs
RMSD: root-mean-square deviation
RNA-seq: whole transcriptome sequencing
ROC curve: Receiver operator characteristic curves
TMAP: Torrent Mapping Alignment Program
UHRR: Agilent Universal Human Reference RNA

## References

[1] Love MI, Huber W, Anders S (2014). Moderated estimation of fold change and dispersion for RNA-seq data with DESeq2. Genome Biol.

[2] Yaari G, Bolen CR, Thakar J, Kleinstein SH (2013). Quantitative set analysis for gene expression: a method to quantify gene set differential expression including gene-gene correlations. Nucleic Acids Res.

[3] Wang Z, Gerstein M, Snyder M (2009). RNA-Seq: a revolutionary tool for transcriptomics. Nat Rev Genet.

[4] Clark TA, Sugnet CW, Ares M (2002). Genomewide analysis of mRNA processing in yeast using splicing-specific microarrays. Science.

[5] David L, Huber W, Granovskaia M, Toedling J, Palm CJ, Bofkin L (2006). A high-resolution map of transcription in the yeast genome. Proc Natl Acad Sci U S A.

[6] Bertone P, Stolc V, Royce TE, Rozowsky JS, Urban AE, Zhu X (2004). Global identification of human transcribed sequences with genome tiling arrays. Science.

[7] Yamada K, Lim J, Dale JM, Chen H, Shinn P, Palm CJ (2003). Empirical analysis of transcriptional activity in the Arabidopsis genome. Science.

[8] Okoniewski MJ, Miller CJ (2006). Hybridization interactions between probesets in short oligo microarrays lead to spurious correlations. BMC Bioinformatics.

[9] Royce TE, Rozowsky JS, Gerstein MB (2007). Toward a universal microarray: prediction of gene expression through nearest-neighbor probe sequence identification. Nucleic Acids Res.

[10] Zhang JD, Schindler T, Kung E, Ebeling M, Certa U (2014). Highly sensitive amplicon-based transcript quantification by semiconductor sequencing. BMC Genomics.

[11] Canales RD, Luo Y, Willey JC, Austermiller B, Barbacioru CC, Boysen C (2006). Evaluation of DNA microarray results with quantitative gene expression platforms. Nat Biotechnol.

[12] Bradford JR, Hey Y, Yates T, Li Y, Pepper SD, Miller CJ (2010). A comparison of massively parallel nucleotide sequencing with oligonucleotide microarrays for global transcription profiling. BMC Genomics.

[13] Oshlack A, Robinson MD, Young MD (2010). From RNA-seq reads to differential expression results. Genome Biol.

[14] Agarwal A, Koppstein D, Rozowsky J, Sboner A, Habegger L, Hillier LW (2010). Comparison and calibration of transcriptome data from RNA-Seq and tiling arrays. BMC Genomics.

[15] Blomquist TM, Crawford EL, Lovett JL, Yeo J, Stanoszek LM, Levin A (2013). Targeted RNA-sequencing with competitive multiplex-PCR amplicon libraries. PLoS One.

[16] AmpliSeq: https://tools.thermofisher.com/content/sfs/brochures/AmpliSeq-Transcriptome-app-note.pdf. Accessed date on Dec. 11.

[17] Li S, Tighe SW, Nicolet CM, Grove D, Levy S, Farmerie W (2014). Multi-platform assessment of transcriptome profiling using RNA-seq in the ABRF next-generation sequencing study. Nat Biotechnol.

[18] Liu L, Li Y, Li S, Hu N, He Y, Pong R (2012). Comparison of next-generation sequencing systems. J Biomed Biotechnol.

[19] Merriman B, Ion Torrent R, Team D, Rothberg JM (2012). Progress in ion torrent semiconductor chip based sequencing. Electrophoresis.

[20] Consortium M, Shi L, Reid LH, Jones WD, Shippy R, Warrington JA (2006). The MicroArray Quality Control (MAQC) project shows inter- and intraplatform reproducibility of gene expression measurements. Nat Biotechnol.

[21] Consortium SM-I, Consortium SM-I (2014). A comprehensive assessment of RNA-seq accuracy, reproducibility and information content by the Sequencing Quality Control Consortium. Nat Biotechnol.

[22] Rapaport F, Khanin R, Liang Y, Pirun M, Krek A, Zumbo P (2013). Comprehensive evaluation of differential gene expression analysis methods for RNA-seq data. Genome Biol.

[23] Baker SC, Bauer SR, Beyer RP, Brenton JD, Bromley B, Burrill J (2005). The External RNA Controls Consortium: a progress report. Nat Methods.

[24] Arnett DK, Devereux RB, Kitzman D, Oberman A, Hopkins P, Atwood L (2001). Linkage of left ventricular contractility to chromosome 11 in humans: The HyperGEN Study. Hypertension.

[25] Investigators F (2002). Multi-center genetic study of hypertension: The Family Blood Pressure Program (FBPP). Hypertension.

[26] Yu J, Hu K, Smuga-Otto K, Tian S, Stewart R, Slukvin II (2009). Human induced pluripotent stem cells free of vector and transgene sequences. Science.

[27] Ma J, Guo L, Fiene SJ, Anson BD, Thomson JA, Kamp TJ (2011). High purity human-induced pluripotent stem cell-derived cardiomyocytes: electrophysiological properties of action potentials and ionic currents. Am J Physiol Heart Circ Physiol.

[28] Aggarwal P, Turner A, Matter A, Kattman SJ, Stoddard A, Lorier R (2014). RNA expression profiling of human iPSC-derived cardiomyocytes in a cardiac hypertrophy model. PLoS One.

[29] Kim D, Pertea G, Trapnell C, Pimentel H, Kelley R, Salzberg SL (2013). TopHat2: accurate alignment of transcriptomes in the presence of insertions, deletions and gene fusions. Genome Biol.

[30] Langmead B, Salzberg SL (2012). Fast gapped-read alignment with Bowtie 2. Nat Methods.

[31] Anders S, Pyl PT, Huber W (2015). HTSeq--a Python framework to work with high-throughput sequencing data. Bioinformatics.

[32] Li H, Durbin R (2009). Fast and accurate short read alignment with Burrows-Wheeler transform. Bioinformatics.

[33] Li H, Durbin R (2010). Fast and accurate long-read alignment with Burrows-Wheeler transform. Bioinformatics.

[34] Ning Z, Cox AJ, Mullikin JC (2001). SSAHA: a fast search method for large DNA databases. Genome Res.

[35] Li H (2012). Exploring single-sample SNP and INDEL calling with whole-genome de novo assembly. Bioinformatics.

[36] Smith TF, Waterman MS (1981). Identification of common molecular subsequences. J Mol Biol.

[37] TMAP: http://mendel.iontorrent.com/ion-docs/Technical-Note---TMAP-Alignment_9012907.html. Accessed date on Dec. 11.

[38] Pedregosa F, Varoquaux G, Gramfort A, Michel V, Thirion B, Grisel O (2011). Scikit-learn: Machine Learning in Python. J Mach Learn Res.

[39] Ritchie ME, Phipson B, Wu D, Hu Y, Law CW, Shi W (2015). limma powers differential expression analyses for RNA-sequencing and microarray studies. Nucleic Acids Res.

[40] Rau A, Marot G, Jaffrezic F (2014). Differential meta-analysis of RNA-seq data from multiple studies. BMC Bioinformatics.

[41] Li B, Ruotti V, Stewart RM, Thomson JA, Dewey CN (2010). RNA-Seq gene expression estimation with read mapping uncertainty. Bioinformatics.

[42] Liang P (2007). MAQC papers over the cracks. Nat Biotechnol.

[43] Shippy R, Fulmer-Smentek S, Jensen RV, Jones WD, Wolber PK, Johnson CD (2006). Using RNA sample titrations to assess microarray platform performance and normalization techniques. Nat Biotechnol.

[44] Wang C, Gong B, Bushel PR, Thierry-Mieg J, Thierry-Mieg D, Xu J (2014). The concordance between RNA-seq and microarray data depends on chemical treatment and transcript abundance. Nat Biotechnol.

[45] Binsheng Gong CW, Zhenqiang Su, Huixiao HOng, Jean Thierry-Mieg, Danielle Thierry-Mieg, Leming Shi, et al: Transcriptomic profiling of rat liver samples in a comprehensive study design by RNA-Seq. Sci Data 2014; doi:10.1038/sdata.2014.21. 10.1038/sdata.2014.21 PMC4322565 25977778

[46] t Hoen PA, Friedlander MR, Almlof J, Sammeth M, Pulyakhina I, Anvar SY (2013). Reproducibility of high-throughput mRNA and small RNA sequencing across laboratories. Nat Biotechnol.

