# Supplementary figures and images for: Comprehensive evaluation of AmpliSeq transcriptome, a novel targeted whole transcriptome RNA sequencing methodology for global gene expression analysis
Source: BMC Genomics. 2015 Dec 16;16:1069. doi: 10.1186/s12864-015-2270-1 (PMC4681149; doi:10.1186/s12864-015-2270-1)

# Spearman's Ranked R

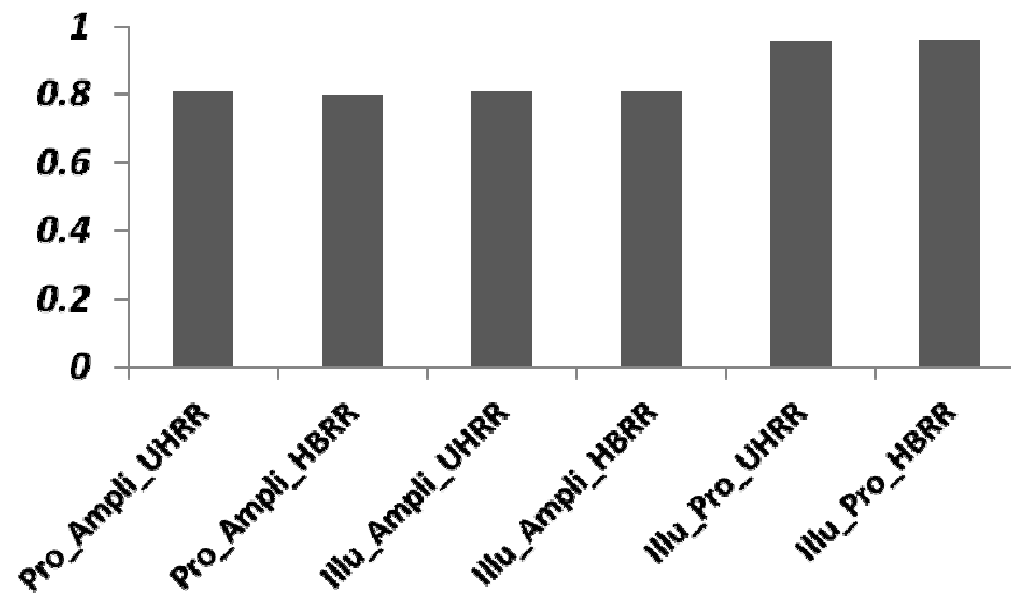

Supp. Figure 2. a

Supplement: Additional file 1: Figure S1. — Genes with at least two-fold change in expression between UHRR and HBRR have a nearly even distribution in four quartiles in terms of transcript abundance based on normalized transcript read-counts from Illumina RNA-seq. Figure S2. a.) Spearman’s ranked r for all genes using log10 transformed read-counts. AmpliSeq showed a strong correlation to the two whole transcriptome RNA-seq methods as determined by Spearman’s ranked r. b.) Dotplots of gene expression between different sequencing platforms. Figure S3. Significant correlation (p < 1e-6) of gene expression (using log10 transformed read-counts) between AmpliSeq and Proton RNA-seq for the following samples: hiPSC-CM 1104 at stimulated condition, hiPSC-CM 1104 at unstimulated condition, hiPSC-CM 1156 at stimulated condition and hiPSC-CM 1156 at unstimulated condition (E: Endothelin 1 stimulated, U: unstimulated). Figure S4. Significant correlation (p < 1e-6) of log2FC between AmpliSeq and Proton RNA-seq for samples hiPSC-CM 1156 (ET vs. unstim, n = 10,183) and hiPSC-CM 1104 (ET. vs. unstim, n = 10,226). Figure S5. All three methods show strong correlation against the RT-qPCR results in terms of log2FC. Using the MAQC dataset as the standard, we observe Pearson’s r values of 0.95 between the log2FC determined by AmpliSeq and the two RNA-seq methods (n = 674). For the ABRF PrimePCR dataset, the Pearson’s values were > =0.89 (n = 13,747). (ZIP 295 kb) [file 12864_2015_2270_MOESM1_ESM.zip › AddF2.pdf]

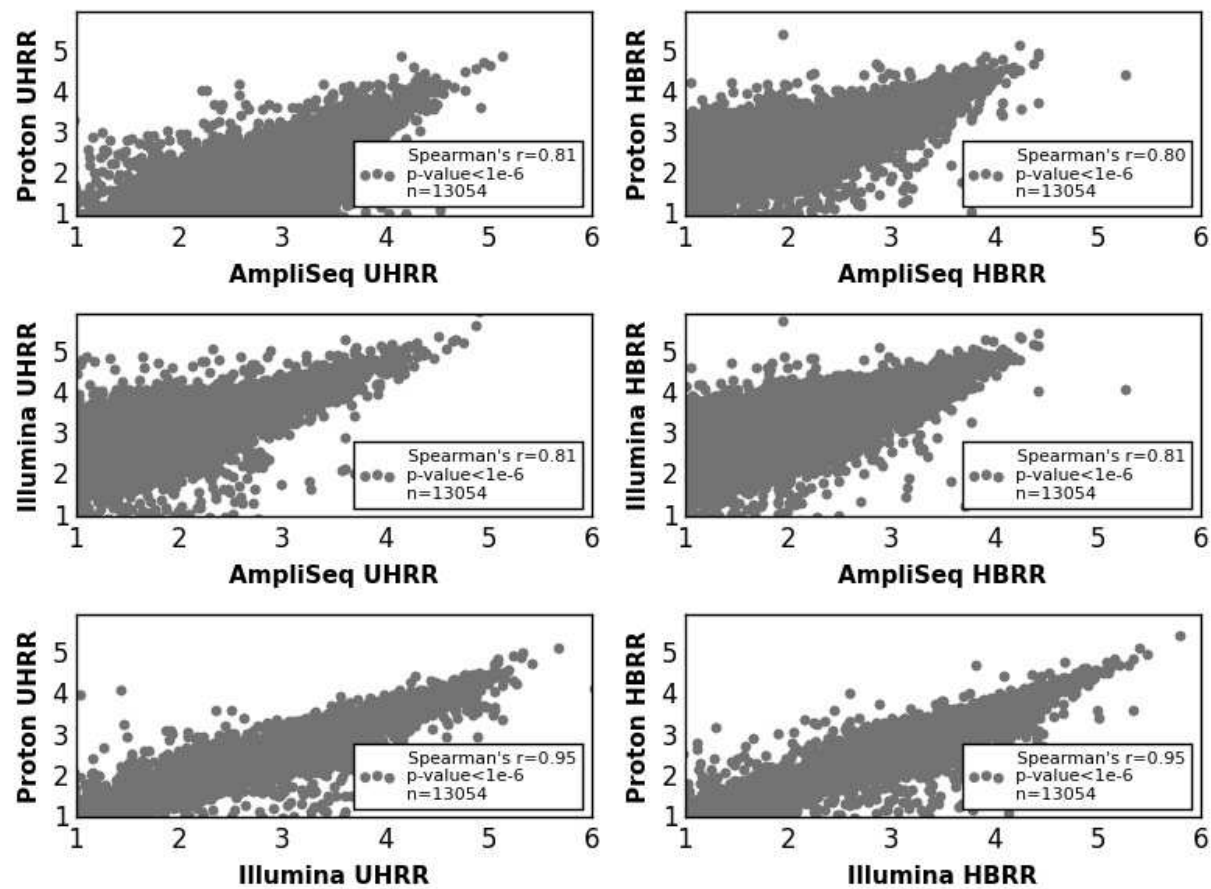

Supp. Figure 2. b

Supplement: Additional file 1: Figure S1. — Genes with at least two-fold change in expression between UHRR and HBRR have a nearly even distribution in four quartiles in terms of transcript abundance based on normalized transcript read-counts from Illumina RNA-seq. Figure S2. a.) Spearman’s ranked r for all genes using log10 transformed read-counts. AmpliSeq showed a strong correlation to the two whole transcriptome RNA-seq methods as determined by Spearman’s ranked r. b.) Dotplots of gene expression between different sequencing platforms. Figure S3. Significant correlation (p < 1e-6) of gene expression (using log10 transformed read-counts) between AmpliSeq and Proton RNA-seq for the following samples: hiPSC-CM 1104 at stimulated condition, hiPSC-CM 1104 at unstimulated condition, hiPSC-CM 1156 at stimulated condition and hiPSC-CM 1156 at unstimulated condition (E: Endothelin 1 stimulated, U: unstimulated). Figure S4. Significant correlation (p < 1e-6) of log2FC between AmpliSeq and Proton RNA-seq for samples hiPSC-CM 1156 (ET vs. unstim, n = 10,183) and hiPSC-CM 1104 (ET. vs. unstim, n = 10,226). Figure S5. All three methods show strong correlation against the RT-qPCR results in terms of log2FC. Using the MAQC dataset as the standard, we observe Pearson’s r values of 0.95 between the log2FC determined by AmpliSeq and the two RNA-seq methods (n = 674). For the ABRF PrimePCR dataset, the Pearson’s values were > =0.89 (n = 13,747). (ZIP 295 kb) [file 12864_2015_2270_MOESM1_ESM.zip › AddF2b.pdf]

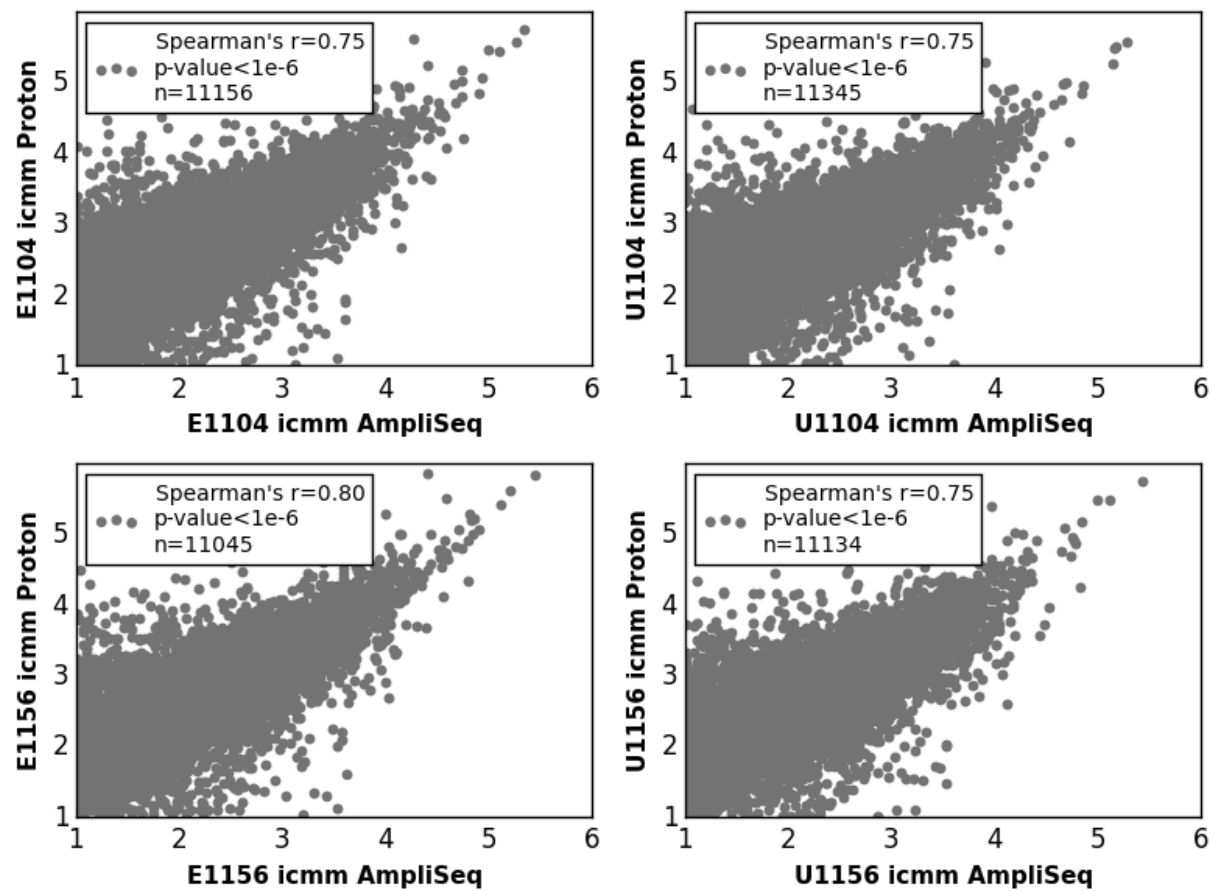

**Supp. Figure 3**

Supplement: Additional file 1: Figure S1. — Genes with at least two-fold change in expression between UHRR and HBRR have a nearly even distribution in four quartiles in terms of transcript abundance based on normalized transcript read-counts from Illumina RNA-seq. Figure S2. a.) Spearman’s ranked r for all genes using log10 transformed read-counts. AmpliSeq showed a strong correlation to the two whole transcriptome RNA-seq methods as determined by Spearman’s ranked r. b.) Dotplots of gene expression between different sequencing platforms. Figure S3. Significant correlation (p < 1e-6) of gene expression (using log10 transformed read-counts) between AmpliSeq and Proton RNA-seq for the following samples: hiPSC-CM 1104 at stimulated condition, hiPSC-CM 1104 at unstimulated condition, hiPSC-CM 1156 at stimulated condition and hiPSC-CM 1156 at unstimulated condition (E: Endothelin 1 stimulated, U: unstimulated). Figure S4. Significant correlation (p < 1e-6) of log2FC between AmpliSeq and Proton RNA-seq for samples hiPSC-CM 1156 (ET vs. unstim, n = 10,183) and hiPSC-CM 1104 (ET. vs. unstim, n = 10,226). Figure S5. All three methods show strong correlation against the RT-qPCR results in terms of log2FC. Using the MAQC dataset as the standard, we observe Pearson’s r values of 0.95 between the log2FC determined by AmpliSeq and the two RNA-seq methods (n = 674). For the ABRF PrimePCR dataset, the Pearson’s values were > =0.89 (n = 13,747). (ZIP 295 kb) [file 12864_2015_2270_MOESM1_ESM.zip › AddF3.pdf]

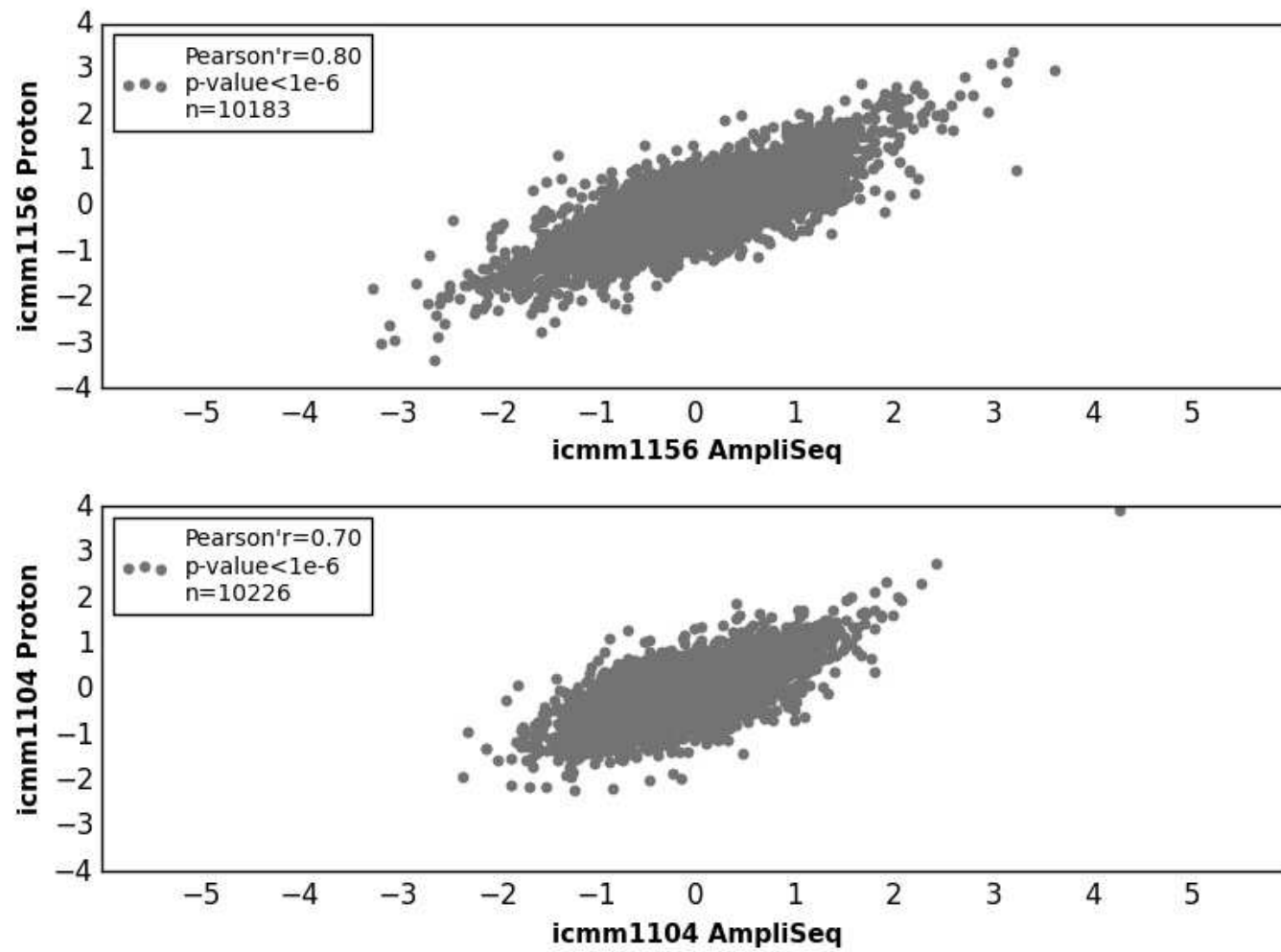

Supp. Figure 4

Supplement: Additional file 1: Figure S1. — Genes with at least two-fold change in expression between UHRR and HBRR have a nearly even distribution in four quartiles in terms of transcript abundance based on normalized transcript read-counts from Illumina RNA-seq. Figure S2. a.) Spearman’s ranked r for all genes using log10 transformed read-counts. AmpliSeq showed a strong correlation to the two whole transcriptome RNA-seq methods as determined by Spearman’s ranked r. b.) Dotplots of gene expression between different sequencing platforms. Figure S3. Significant correlation (p < 1e-6) of gene expression (using log10 transformed read-counts) between AmpliSeq and Proton RNA-seq for the following samples: hiPSC-CM 1104 at stimulated condition, hiPSC-CM 1104 at unstimulated condition, hiPSC-CM 1156 at stimulated condition and hiPSC-CM 1156 at unstimulated condition (E: Endothelin 1 stimulated, U: unstimulated). Figure S4. Significant correlation (p < 1e-6) of log2FC between AmpliSeq and Proton RNA-seq for samples hiPSC-CM 1156 (ET vs. unstim, n = 10,183) and hiPSC-CM 1104 (ET. vs. unstim, n = 10,226). Figure S5. All three methods show strong correlation against the RT-qPCR results in terms of log2FC. Using the MAQC dataset as the standard, we observe Pearson’s r values of 0.95 between the log2FC determined by AmpliSeq and the two RNA-seq methods (n = 674). For the ABRF PrimePCR dataset, the Pearson’s values were > =0.89 (n = 13,747). (ZIP 295 kb) [file 12864_2015_2270_MOESM1_ESM.zip › AddF4.pdf]

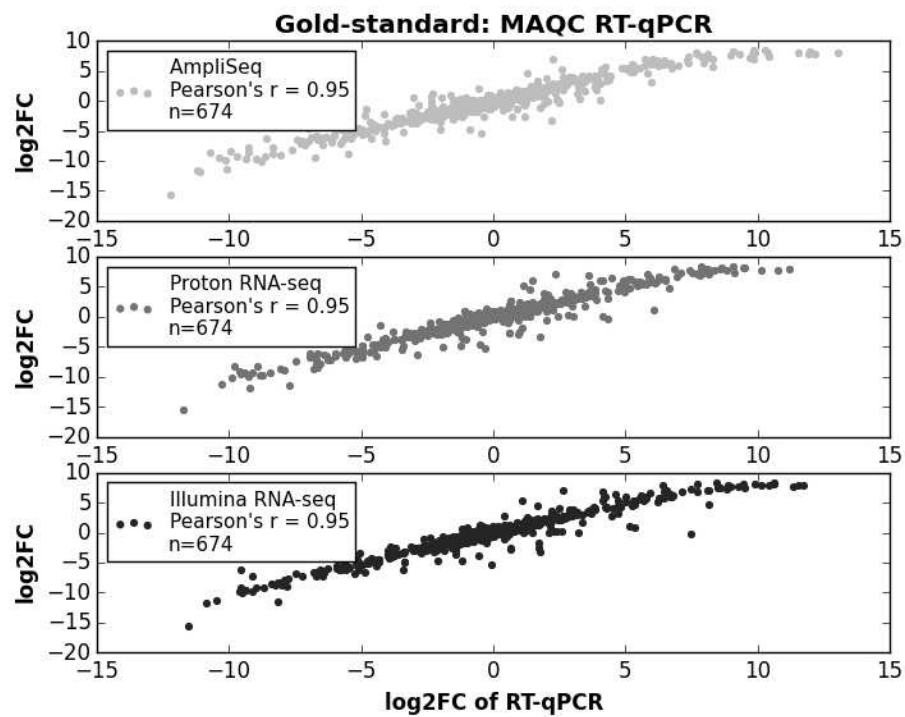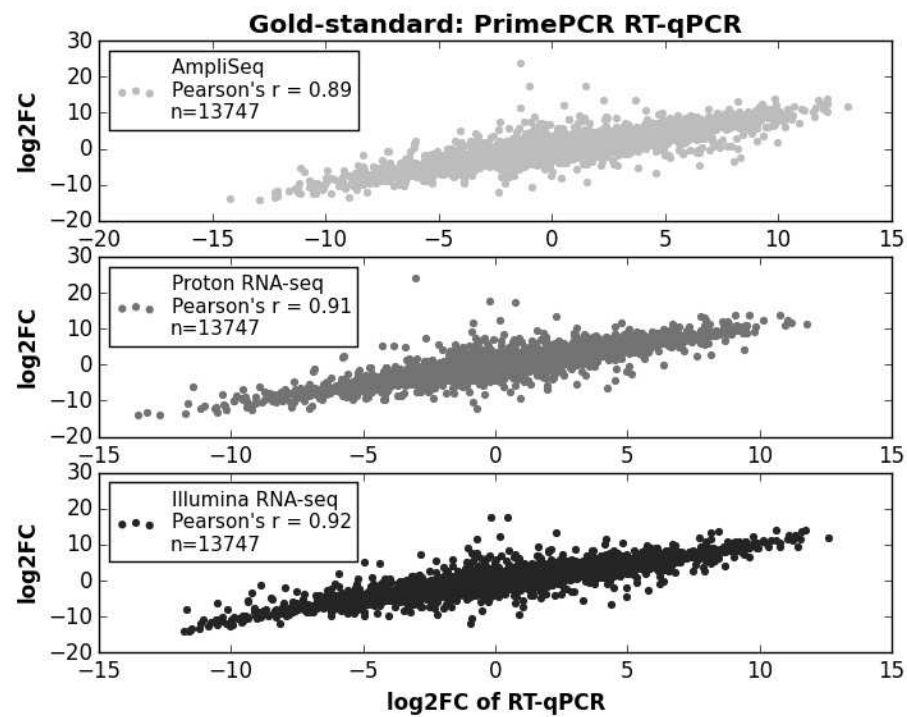

**Supp. Figure 5**

Supplement: Additional file 1: Figure S1. — Genes with at least two-fold change in expression between UHRR and HBRR have a nearly even distribution in four quartiles in terms of transcript abundance based on normalized transcript read-counts from Illumina RNA-seq. Figure S2. a.) Spearman’s ranked r for all genes using log10 transformed read-counts. AmpliSeq showed a strong correlation to the two whole transcriptome RNA-seq methods as determined by Spearman’s ranked r. b.) Dotplots of gene expression between different sequencing platforms. Figure S3. Significant correlation (p < 1e-6) of gene expression (using log10 transformed read-counts) between AmpliSeq and Proton RNA-seq for the following samples: hiPSC-CM 1104 at stimulated condition, hiPSC-CM 1104 at unstimulated condition, hiPSC-CM 1156 at stimulated condition and hiPSC-CM 1156 at unstimulated condition (E: Endothelin 1 stimulated, U: unstimulated). Figure S4. Significant correlation (p < 1e-6) of log2FC between AmpliSeq and Proton RNA-seq for samples hiPSC-CM 1156 (ET vs. unstim, n = 10,183) and hiPSC-CM 1104 (ET. vs. unstim, n = 10,226). Figure S5. All three methods show strong correlation against the RT-qPCR results in terms of log2FC. Using the MAQC dataset as the standard, we observe Pearson’s r values of 0.95 between the log2FC determined by AmpliSeq and the two RNA-seq methods (n = 674). For the ABRF PrimePCR dataset, the Pearson’s values were > =0.89 (n = 13,747). (ZIP 295 kb) [file 12864_2015_2270_MOESM1_ESM.zip › AddF5.pdf]
